# Planar Zn-Ion Microcapacitors with High-Capacity Activated Carbon Anode and VO_2_ (B) Cathode

**DOI:** 10.1021/acs.nanolett.4c02539

**Published:** 2024-08-20

**Authors:** Yujia Fan, Iman Pinnock, Xueqing Hu, Tianlei Wang, Yinan Lu, Ruixiang Li, Mingqing Wang, Ivan P. Parkin, Michael De Volder, Buddha Deka Boruah

**Affiliations:** † Institute for Materials Discovery, University College London, London WC1E 7JE, United Kingdom; ‡ Department of Chemistry, University College London, London, WC1H 0AJ, U.K.; § School of Engineering and Materials Science, Queen Mary University of London, London, E1 4NS, U.K.; ∥ Institute for Manufacturing, University of Cambridge, Cambridge, CB3 0FS, U.K.

**Keywords:** Zn-ion microcapacitors, high-capacity materials, dendrite-free electrodes, effective mass loading

## Abstract

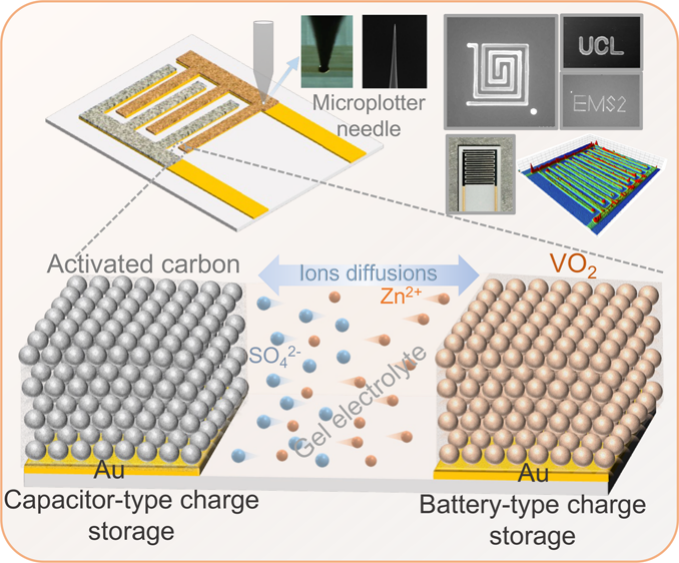

The downsizing of microscale energy storage devices plays a crucial role in powering modern emerging devices. Therefore, the scientific focus on developing high-performance microdevices, balancing energy density and power density, becomes essential. In this context, we explore an advanced Microplotter technique to fabricate hybrid planar Zn-ion microcapacitors (ZIMCs) that exhibit dual charge storage characteristics, with an electrical double layer capacitor type activated carbon anode and a battery type VO_2_ (B) cathode, aiming to achieve energy density surpassing supercapacitors and power density exceeding batteries. Effective loading of VO_2_ (B) cathode electrode materials combined with activated carbon anode onto confined planar microelectrodes not only provides reversible Zn^2+^ storage performance but also mitigates dendrite formation. This not only results in superior charge storage performance, including areal energies of 2.34 μWh/cm^2^ (at 74.76 μW/cm^2^) and 0.94 μWh/cm^2^ (at 753.12 μW/cm^2^), exceeding performance of zinc nanoparticle anode and activated carbon cathode based ZIMCs, but also ensures stable capacity retention of 87% even after 1000 cycles and free from any unwanted dendrites. Consequently, this approach is directed toward the development of high-performance ZIMCs by exploring high-capacity materials for efficient utilization on microelectrodes and achieving maximum possible capacities within the constraints of the limited device footprint.

Microelectronic devices designed for wearables and implants, such as microrobots and microsensors, have made remarkable progress and are on the verge of becoming integral parts of our daily lives. These compact devices excel in intricate tasks like data processing and wireless signal transmission within a space smaller than a few cubic millimeters, holding immense potential in fields like health monitoring, medical diagnosis, and disease treatment.^1^ For these devices to operate, a crucial component is the energy supply unit. The ongoing trend of reducing the size of wearable and implantable microelectronics while enhancing their capabilities necessitates corresponding micropower sources. These include microscale energy storage devices capable of delivering substantial energy outputs. Conventional microscale energy storage device structures, resembling layered sandwiches with positive and negative electrodes separated by separators, pose various challenges, such as precise electrode alignment and integration issues when seamlessly incorporating them with on-chip microelectronics.^2^ However, an alternative design, known as planar-type device configurations, organizes electrodes in an interdigitated electrode (IDE) pattern on the same substrate, resulting in a flat device structure. This design offers several advantages, including better control over critical battery attributes like internal resistance and ionic diffusion distance, all without the need for a separator.^3^ Most importantly, it provides a practical solution for reducing battery size and seamlessly integrating them with on-chip microelectronic devices. Therefore, the recent development of high-performance planar microscale energy storage devices has garnered interest. For instance, hybrid microcapacitors exhibit energy storage characteristics that surpass the charge storage properties of supercapacitors, while simultaneously offering power density exceeding that of traditional batteries, along with long-term cycling stability.^4^

Hybrid microcapacitors, specifically planar zinc-ion microcapacitors (ZIMCs), offer additional advantages such as cost-effectiveness and abundance. These devices exhibit superior energy density compared to supercapacitors and higher power density than zinc-ion batteries. Among the various high-capacity electrode materials explored for hybrid Zn-ion capacitors, carbon-based cathodes and metal Zn anodes have been widely investigated.^5−7^ These configurations provide enhanced charge storage performance, attributed to the high surface area of activated carbon (1000–1500 m^2^/g) and the high theoretical capacity of the Zn metal anode (820 mAh/g). In ZIMCs, the electrode configuration involving Zn electrodeposition on conducting IDEs has been successfully applied. For instance, Sun et al. utilized Zn electrodeposition on predesigned carbon nanotube-based IDEs as the anode against a cathode made of carbon nanotubes.^8^ Similarly, Zhang et al. electrodeposited Zn nanosheets as the anode and applied a slurry containing activated carbon as the cathode onto gold IDEs for ZIMCs.^9^ However, Zn anodes exhibit significant dendrite growth in aqueous electrolytes.^10−12^ To overcome this challenge, an alternative device configuration explores metal-oxide-based cathodes (e.g., V-based, Mn-based, etc.). This configuration is widely applied in conventional coin cell-based Zn-ion capacitors, such as activated carbon anode//V_2_O_5_ cathode,^13^ MXene anode//V_2_O_5_ cathode,^14^ activated carbon anode//MnO_2_ cathode,^15^ MXene anode//MnO_2_–carbon nanotubes cathod,^16^ activated carbon anode//Zn_*x*_MnO_2_ cathode,^17^ TiS_2_ anode//activated carbon cathode^18^ and so forth. It is noteworthy that loading oxide-based materials onto planar IDEs presents challenges using conventional electrodeposition processes, particularly in increasing the mass loading due to limited conductivity. Nevertheless, commonly employed techniques like screen printing,^19^ 3D printing,^20^ and mask-assisted spray processing^21^ could facilitate loading of oxide and carbon-based materials. However, these methods face difficulties with microelectrode gaps narrower than 300 μm. Therefore, it becomes crucial to explore alternative techniques that enable effective loading/deposition of high-capacity cathodes along with high-capacity carbon-based anode materials onto microelectrodes to achieve high-performance and dendrite-free ZIMCs.

This research specifically explores the application of high-capacity materials, utilizing an activated carbon anode and VO_2_ (B) cathode for ZIMC, followed by the higher resolution and precise loading of these materials onto confined metal IDEs with a 200 μm electrode gap by using the advanced Microplotter technique. As anticipated, the use of the combination of high-capacity battery-type VO_2_ (B) cathode material and capacitor-type activated carbon (AC) anode material not only delivers impressive charge storage performance, including peak an areal energy of 2.34 μWh/cm^2^ and areal power of 753.12 μW/cm^2^, than Zn nanoparticles anode//AC cathode based ZIMCs (areal energy of 1.25 μWh/cm^2^) but also ensures dendrite-free performance even during long-term cycling of 1000 cycles and remarkable capacity retention of 87%. As a result, this research has the potential to introduce a potential approach for integrating high-capacity materials onto microelectrodes, thereby advancing the development of high-performance microscale energy storage devices.

We employed the Microplotter technique to fabricate planar zinc-ion microcapacitors (ZIMCs). This involved loading electrode ink through the capillarity phenomenon and employing ink dropping, driven by the piezoelectric vibration of the nozzle. The primary focus was on precise and effective loading of electrode materials onto gold (Au, 4 μm thick) interdigitated electrodes (IDEs). While printing active materials, the software automatically arranged the line spacing, maintaining a line width of 50 μm, in accordance with the feature width of Au IDEs. Figure 1a outlines the fabrication process for ZIMCs, involving the patterning of Au IDEs on a ceramic substrate, followed by the loading of activated carbon (AC) as the anode and VO_2_ (B) as the cathode for the AC//VO_2_ ZIMCs. The synthesis details of the electrode materials are provided in the Experimental Section in the Supporting Information. Figure 1b presents a digital image of an AC//VO_2_ ZIMC. The ZIMCs were tested by using a gel electrolyte containing 1 M ZnSO_4_ in a gelatin matrix (refer to the experimental section for preparation details). Our planar-type ZIMCs exhibit a distinct ion diffusion mechanism compared to conventional sandwich-type devices. Specifically, in-plane ion diffusion takes place, as depicted in Figures 1c, where capacitor-type charge storage (adsorption/desorption of electrolyte ions) occurs on the AC anode, and battery-type processes (intercalation/dentercalation) take place on the VO_2_ (B) cathode in AC//VO_2_ ZIMCs. The unique utilization of the Microplotter technique (Figure 1d) enables the direct printing of diverse materials on target substrates with pattern sizes controlled by ink viscosity, particle sizes, and the use of a glass needle (Figure 1e). For instance, Figure 1g-h shows Scanning Electron Microscopy (SEM) images illustrating different patterns directly printed using the Microplotter, including rectangular nested concentric patterns, as well as direct letter writing onto substrates. The delicate viscosity balance in optimizing the ink of electrode materials, including AC, VO_2_ (B), and zinc (Zn), as detailed in the experimental section, is crucial for effective loading onto the desired IDE current collectors. Through careful optimization, testing, and printing sequences, the processing of ZIMCs is precisely refined. Figures 1i and j provide a 2D and 3D representation of the essence of AC//VO_2_ ZIMCs obtained using the profilometer technique. These images highlight the successful loading of electrode materials onto the respective Au IDEs (below is the image of the respective height profiles of the microelectrodes) without issues such as ink leakage or short circuits. This is further confirmed by the SEM image of the AC//VO_2_ ZIMC (Figure 1k), affirming the uniformity and consistent distribution of AC on the anode and VO_2_ (B) on the cathode across the Au IDEs. To maintain standard electrode material composition, the active materials (e.g., AC or VO_2_ (B)) were mixed with SuperP as a conductive additive (see further details in the experimental section). Top-view SEM images of the microelectrodes confirm the distribution of SuperP particles with AC particles (Figures 1l, S1a) and VO_2_ (B) nanowires (Figures 1m, S1b), where the glimpse into the morphologies of these electrode materials is discussed in a later section. Moving beyond surface analysis, the SEM investigation extends to cross-sectional images in Figure 1n (AC anode) and Figure 1o (VO_2_ (B) cathode), respectively.

**Figure 1:**
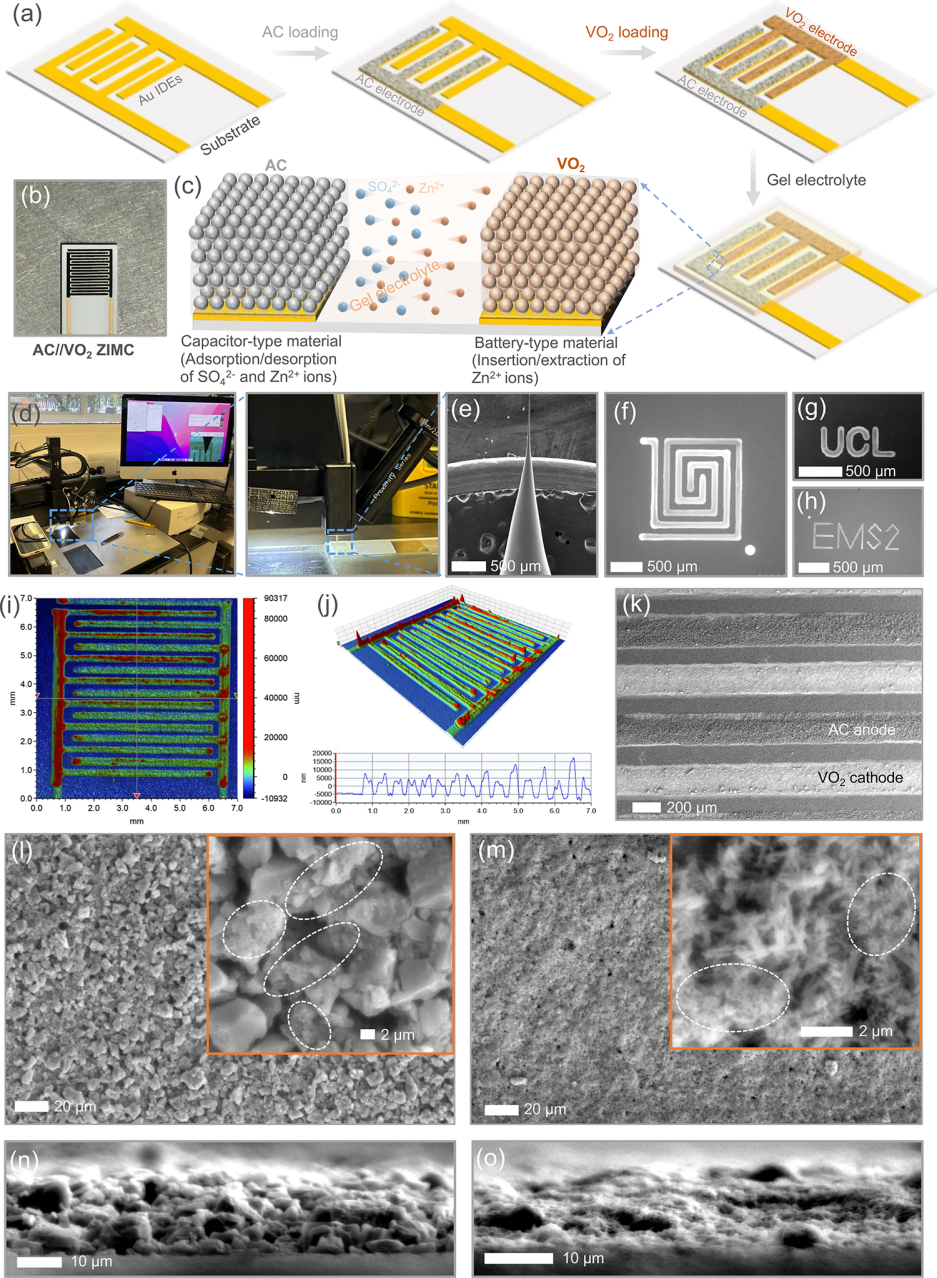
(a) Sequential stages in the fabrication of AC//VO_2_ ZIMCs, illustrating the patterning of Au IDEs with a 200 μm gap on an insulating substrate, followed by the sequential loading of AC anode and VO_2_ (B) cathode onto the Au IDEs using the Microplotter technique, and subsequent testing in gel electrolyte. (b) Digital representation of an AC//VO_2_ ZIMC. (c) Magnified view of a section of the planar AC//VO_2_ ZIMC illustrating in-plane diffusion of electrolyte ions for charge storage: capacitive type onto AC anode and battery type onto VO_2_(B) cathode. (d) Digital images of the Microplotter and (e) SEM image of the glass needle used for printing the electrode materials inks. (f) – (h) SEM images showcasing different patterns directly printed, including rectangular nested concentric patterns, capable of direct letters writing onto substrates using the Microplotter. (i) 2D and (j) 3D profilometer mappings of a developed AC//VO_2_ ZIMC. (k) SEM image of the AC//VO_2_ ZIMC, demonstrating successful loading of AC and VO_2_ (B) onto Au IDEs. Below image represents height profiles of the microelectrodes. (l) SEM image of the AC anode (top view) with an inset showing the presence of SuperP (circles marked), used as conductive active material with the AC particles. (m) Top-view SEM image of the VO_2_ (B) cathode, with an inset demonstrating the distribution of VO_2_ (B) nanowires with SuperP particles. (n, o) Cross-sectional SEM images of AC anode and VO_2_ (B) cathode.

Subsequently, we conducted a detailed examination of the as-synthesized VO_2_ (B) cathode materials, with comprehensive materials characterization outlined in the Experimental Section. SEM and transmission electron microscopy (TEM) images of VO_2_ (B), presented in Figure 2a and 2b, validate the nanorod-like morphologies, exhibiting diameters ranging from 80 to 120 nm and an interplanar spacing of approximately 0.58 nm (Figure 2c). This spacing corresponds to the (200) planes of the VO_2_ (B) monoclinic structure, and the phase identification is confirmed by the XRD pattern, according to the standard PDF#81-2392. The observed XRD peaks aligned with the respective planes in Figure 2d further affirm the VO_2_ (B) monoclinic structure with a space group of *C*2/*m*.^22^ Additionally, we performed X-ray photoelectron spectroscopy (XPS) to further analyze the materials. Figure 2e and 2f present the XPS results. The V 2p spectrum (Figure 2e) displays two regions, V 2p_1/2_ and V 2p_3/2_, where peaks for V^4+^ (516.57 and 524.17 eV) and V^5+^ (517.29 and 524.89 eV) are identified after curve fitting. The total area ratio of V^4+^ to V^5+^ peaks is 3.5:1, indicating that 79.9% of vanadium is in the V^4+^ state. In the O 1s spectrum (Figure 2f), a significant peak around 530.00 eV corresponds to lattice oxygen, while an additional peak around 531.29 eV is attributed to adsorbed oxygen. Nevertheless, detailed characterization of the employed AC and Zn nanoparticles is available in the Supporting Information (Figures S2 and S3). The utilized AC particles exhibit a diverse range of sizes (Figure S2a), with the calculated specific surface area being ∼1586 m^2^/g (Figure S2b). This characteristic is especially advantageous for enhancing electrical double-layer capacitance, a phenomenon that significantly contributes to boosting the energy storage performance of the ZIMCs. The X-ray diffraction (XRD) pattern of AC (Figure S2c) reveals characteristic peaks centered at approximately 2θ = 22° and 43.4°, corresponding to the reflections of the (002) and (101) facets, while the broadening of these peaks indicates that AC is in an amorphous state. Moreover, the Raman spectrum of AC (Figure S 2d) exhibits two prominent peaks at approximately 1340 and 1596 cm^–1^, corresponding to the D band and G band. The D band is indicative of lattice defects, edge imperfections, unkempt alignment, and a low-symmetry graphitic structure in AC, while the G band signifies the presence of C=C stretching vibrations found in graphitic carbon regions characterized by sp^2^ hybridized carbon systems. Additionally, two supplementary peaks at higher wavenumbers, namely ∼2682 cm^–1^ (2D) and ∼2907 cm^–1^ (S3), are observed, associated with the overtone of carbon and the presence of few-layered carbon material, further affirming the graphitic nature of the AC material.^23^Figure S3a,b display the SEM images of the employed Zn nanoparticles, with sizes ranging from 40 to 60 nm, utilized as the anode against the AC cathode in Zn//AC ZIMC. A comparison of the results is made with the AC//VO_2_ ZIMC, where AC serves as the anode while VO_2_ (B) acts as the cathode (see further details). Figure S3c illustrates the XRD pattern of the Zn powder, with characteristic peaks corresponding to the (002), (100), (101), and (102) planes of hexagonal Zn, referring to standard PDF#87-0713. Figure S3d presents the Zn 2p XPS spectra for zinc powder, displaying Zn 2p_1/2_ at 1046.2 eV and Zn 2p_3/2_ at 1023.2 eV.

**Figure 2:**
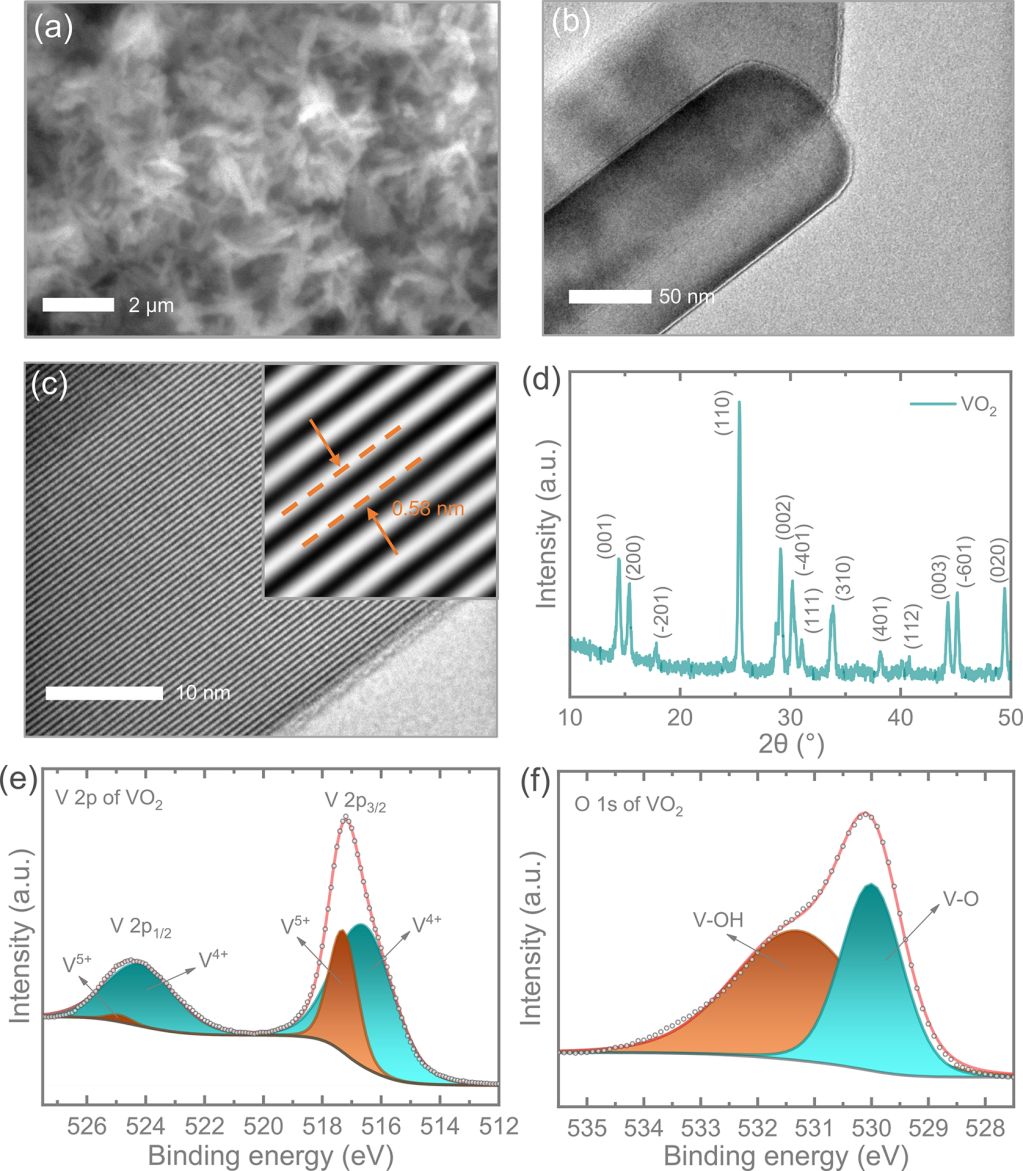
(a) SEM image illustrating the nanowire-like morphology of the synthesized VO_2_ (B) sample, further validated by (b) TEM image. (c) Determination of *d*-spacing, approximately ≈0.58 nm, corresponding to the (200) planes of the monoclinic structure of VO_2_ (B). (d) XRD pattern of VO_2_ (B), confirming its monoclinic VO_2_ (B) phase with a space group of *C*2/*m*. High-resolution (e) V 2p and (f) O 1s XPS spectra of VO_2_ (B).

To enhance the understanding of charge storage performance, we compared the outcomes of Zn//AC ZIMCs (Zn anode and AC cathode) with those of AC//VO_2_ ZIMCs (AC anode and VO_2_ (B) cathode). Figure S4 presents images, including digital images and SEM images, of a Zn//AC ZIMC. For a meaningful comparison, we maintained an identical volume of electrode inks in both sets of ZIMCs (Zn//AC and AC//VO_2_). It is noteworthy that VO_2_ (B) features distinctive tunnel transport pathways, measuring 0.82 nm^2^ along the *b*-axis and 0.5 nm^2^ along the *c*-axis (Figure S5a).^24^ These pathways facilitate effective intercalation and deintercalation of Zn^2+^ ions, enabling the material to achieve reversible capacities exceeding 400 mAh/g. Additionally, VO_2_ (B) exhibits an excellent rate capability. To validate the distinct charge storage performance of VO_2_, we assembled Zn-ion batteries using as-synthesized VO_2_ (B) materials as the cathode against a Zn metal anode. The batteries were tested in a coin cell configuration (CR 2032) using an aqueous electrolyte (see Supporting Information). As illustrated in Figure S5, VO_2_ (B) exhibited impressive specific capacities of 415, 388, 349, 221, and 103 mAh/g at specific currents of 500, 1000, 2000, 5000, and 10000 mA/g. Furthermore, VO_2_ (B) demonstrated a stable charge storage capacity, with, for instance, 94% capacity retention maintained even after 200 charge–discharge cycles, as depicted in Figure S5. Further details are provided in the Supporting Information. Given the remarkable Zn^2+^ ion storage capacities of VO_2_ (B), this material holds potential as a cathode material for realizing high-performance ZIMCs, as elaborated in subsequent sections.

We assessed the charge storage performance of the fabricated ZIMCs in a gel electrolyte by dissolving 1 M ZnSO_4_ in a gelatin matrix to form a gel. The ZIMC devices underwent testing by immersing them directly into a cuvette filled with the gel electrolyte and allowing it to solidify for a few hours before testing, as depicted in a digital image of a ZIMC immersed in an electrolyte for testing (Figure S6). Initially, we conducted cyclic voltamograms (CVs) of the ZIMCs at various scan rates (from 10 mV/s to 500 mV/s, with a voltage range of 0.6 to 1.4 V). Figure 3a illustrates the comparative CV at a scan rate of 100 mV/s, where a significantly higher charge storage performance of AC//VO_2_ ZIMCs was observed compared to that of Zn//AC ZIMCs, with a recorded ∼180% enhancement in the CV area. Furthermore, Figure S7a illustrates the comparative CVs at 500 mV/s, demonstrating that even at higher scan rates, the AC//VO_2_ ZIMCs maintain capacity improvements of ∼165% compared to the Zn//AC ZIMCs. It is noteworthy that AC//VO_2_ ZIMC devices demonstrated a stable potential within the voltage range of 0.6 to 1.4 V, with a slight increase in currents observed toward the lower and higher voltage ends. Additionally, the stable CVs of the AC//VO_2_ ZIMCs at different scan rates, ranging from 10 mV/s to a higher scan rate of 500 mV/s, as shown in Figure 3b, confirm a consistent charge storage response for our AC//VO_2_ ZIMCs. The charge storage mechanism of the AC//VO_2_ ZIMCs aligns with the process of Zn^2+^ cation intercalation onto the VO_2_ (B) cathode for battery-type charge storage, coupled with anions adsorption onto the AC anode for electrical double-layer capacitance-based charge storage during the charging process (Figure 1c). Subsequently, the deintercalation of Zn^2+^ ions from the VO_2_ (B) cathode and the desorption of ions from the AC anode occur during the discharging process. These processes collectively contribute to the charge storage performance of the ZIMCs, with the observed heightened performance attributed to the utilization of a high-capacity VO_2_ (B) cathode for reversible Zn^2+^ ion storage and the efficient electrical double-layer capacitance onto the high-specific surface-area-based AC anodes.

**Figure 3:**
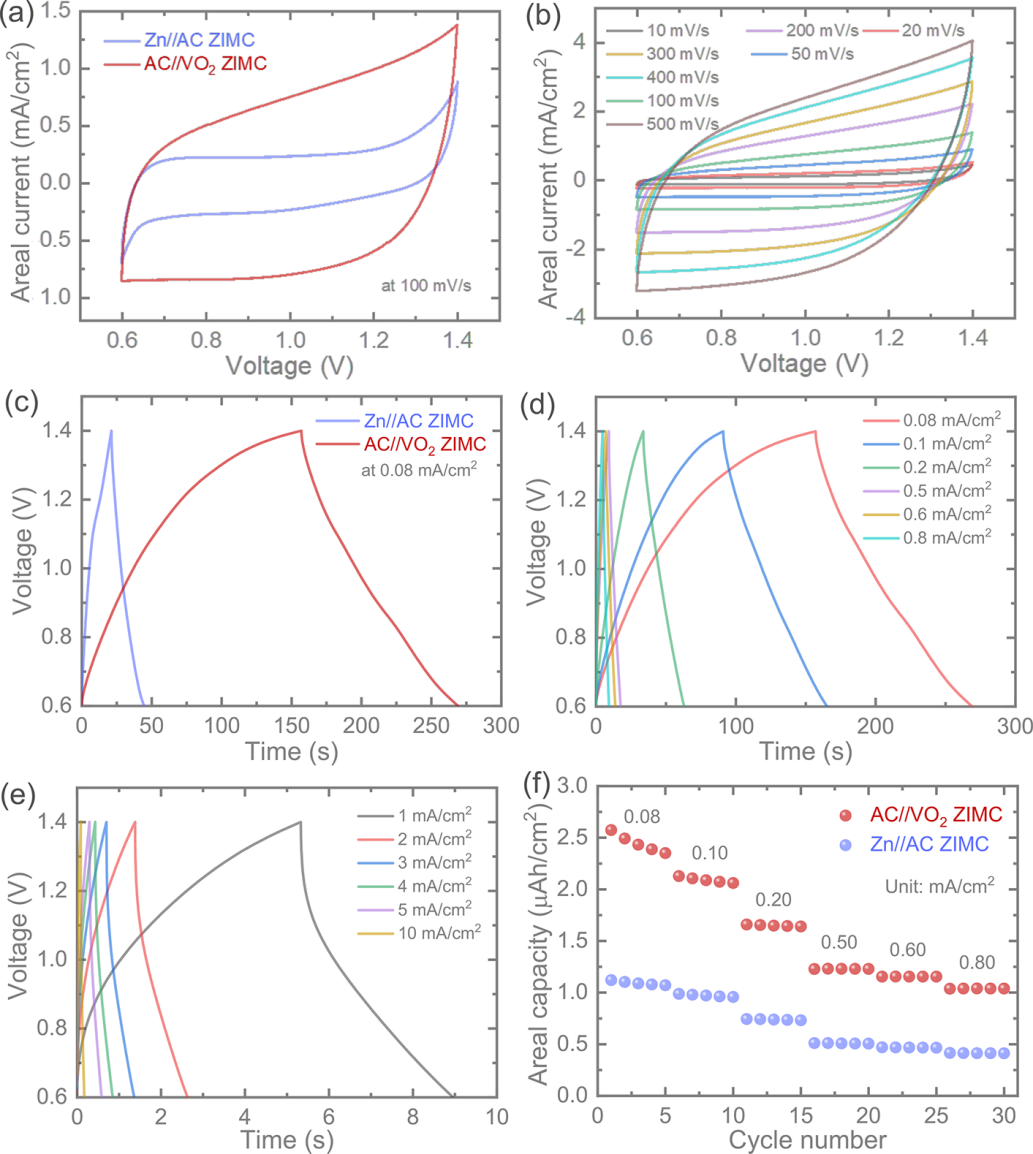
(a) Comparative CVs of Zn//AC and AC//VO_2_ ZIMCs tested at 100 mV/s. (b) CVs of the AC//VO_2_ ZIMC at different scan rates, ranging from 10 to 500 mV/s. (c) GCDs of Zn//AC and AC//VO_2_ ZIMCs tested at 0.08 mA/cm^2^. (d, e) GCD tests of the AC//VO_2_ ZIMC at different areal currents of 0.08 to 0.8 mA/cm^2^ and 1 to 10 mA/cm^2^, respectively. (f) Comparative rate test areal capacity plots of Zn//AC and AC//VO_2_ ZIMCs, highlighting the significantly higher capacity response of AC//VO_2_ ZIMCs compared to Zn//AC ZIMCs.

To gain a deeper understanding of the charge storage performance of the ZIMCs, we extended the electrochemical assessment to galvanostatic charge–discharge (GCD) tests, conducted at various current densities within the same voltage range of 0.6 to 1.4 V employed in CV tests. Consistent with the CV profiles, the comparative GCDs of Zn//AC and AC//VO_2_ ZIMCs tested at 0.08 mA/cm^2^ (Figure 3c) revealed superior charge storage performance in the AC//VO_2_ configuration compared with Zn//AC ZIMCs, aligning with the CV results. Moreover, the AC//VO_2_ ZIMCs exhibited stable GCD profiles across a wide range of areal currents (Figure 3d, e). Even at a very high areal current of 10 mA/cm^2^, AC//VO_2_ ZIMC demonstrated its ability to operate at high rates by delivering an areal capacity of 0.24 μAh/cm^2^. The comparative plot of areal capacities with respect to the areal current for Zn//AC and AC//VO_2_ ZIMCs clearly underscores the superior charge storage performance of the AC//VO_2_ device at each areal current. For instance, the measured areal capacities were 1.01 μAh/cm^2^ (at 0.1 mA/cm^2^), 0.54 μAh/cm^2^ (at 0.5 mA/cm^2^), and 0.42 μAh/cm^2^ (at 0.8 mA/cm^2^) in Zn//AC ZIMCs, which increased to 2.13 μAh/cm^2^, 1.25 μAh/cm^2^, and 1.04 μAh/cm^2^ in AC//VO_2_ ZIMCs, respectively (Figure 3f). Furthermore, the Nyquist plots (Figure S7b and c) are included in the Supporting Information. Notably, the AC//VO_2_ ZIMC demonstrated a lower equivalent series resistance of 7.7 Ω compared to the 11.7 Ω observed in the Zn//AC ZIMC (Figures S7b, c).

Additionally, we computed the areal energies of our Zn//AC and AC//VO_2_ ZIMCs, as illustrated in Figure 4a. Consistent with the observations from Figure 3, we noted superior charge storage performance in the AC//VO_2_ ZIMC compared to Zn//AC. The calculated areal energies were 0.87 μWh/cm^2^, 0.44 μWh/cm^2^, and 0.35 μWh/cm^2^ at areal currents of 0.1 mA/cm^2^, 0.5 mA/cm^2^, and 0.8 mA/cm^2^, respectively, in Zn//AC ZIMCs. These values increased to 1.96 μWh/cm^2^, 1.12 μWh/cm^2^, and 0.94 μWh/cm^2^ in AC//VO_2_ ZIMCs. Furthermore, the Ragone plot (Figure 4b) depicts the energy storage performance in terms of both areal energy and areal power of our AC//VO_2_ ZIMCs, surpassing those of previously reported high-performance microsupercapacitors, including symmetric and asymmetric device configurations. These characteristics further validate the high performance of our AC//VO_2_ ZIMCs. To assess the long-term cycling stability, prolonged cycling tests were conducted on our ZIMCs, as shown in Figure S8. These tests demonstrated capacity retentions of 87% and 81% for AC//VO_2_ and Zn//AC ZIMCs, even after 1000 cycles when tested at 0.1 mA/cm^2^. The relatively low capacity fading of 13% after 1000 cycles for the AC//VO_2_ ZIMC further confirms the charge storage stability. To explore the morphologies of the electrodes after cycling tests, we conducted extended SEM imaging of the cycled electrode materials including VO_2_ (B), AC, and Zn nanoparticles in the ZIMCs. However, we encountered challenges in capturing SEM images of cycled electrodes tested in gel electrolyte, as the gel electrolyte covered the electrode materials and was difficult to clean for SEM imaging (Figure S9). Consequently, for the post-mortem SEM images of the electrode materials, we cycled Zn//AC and AC//VO_2_ ZIMCs in 1 M ZnSO_4_ aqueous electrolyte rather than using ZnSO_4_ gel electrolyte, testing them for 1000 cycles at the same areal current of 0.1 mA/cm^2^ (Figure 4c). Interestingly, even after cycling, the VO_2_ (B) cathode materials maintained identical morphologies without exhibiting any uneven material deposition related to Zn flakes (Figure 4d(i-iii)). However, the Zn anode material showed significant changes in morphologies due to severe Zn flake growth (Figure 4d(iii)). Similar severe Zn flakes growth was also observed in earlier reports when tested in ZIMCs.^25^ The growth of Zn flakes on the Zn nanoparticles anode leads not only to an uneven distribution of electric fields on the electrode surface but also to a short circuit under long-term cycling. In contrast, as anticipated, our VO_2_ (B) electrode materials not only remained free from An flakes/dendrites but also exhibited better charge storage performance of reversible Zn^2+^ intercalation/deintercalation reactions. Additionally, cycled AC electrode materials maintained identical morphologies (Figure 4d(ii)), highlighting the advantages of using dendrite-free and highly stable electrode materials, such as AC anode materials, for high-performance ZIMC applications against VO_2_ (B) cathode materials.

**Figure 4:**
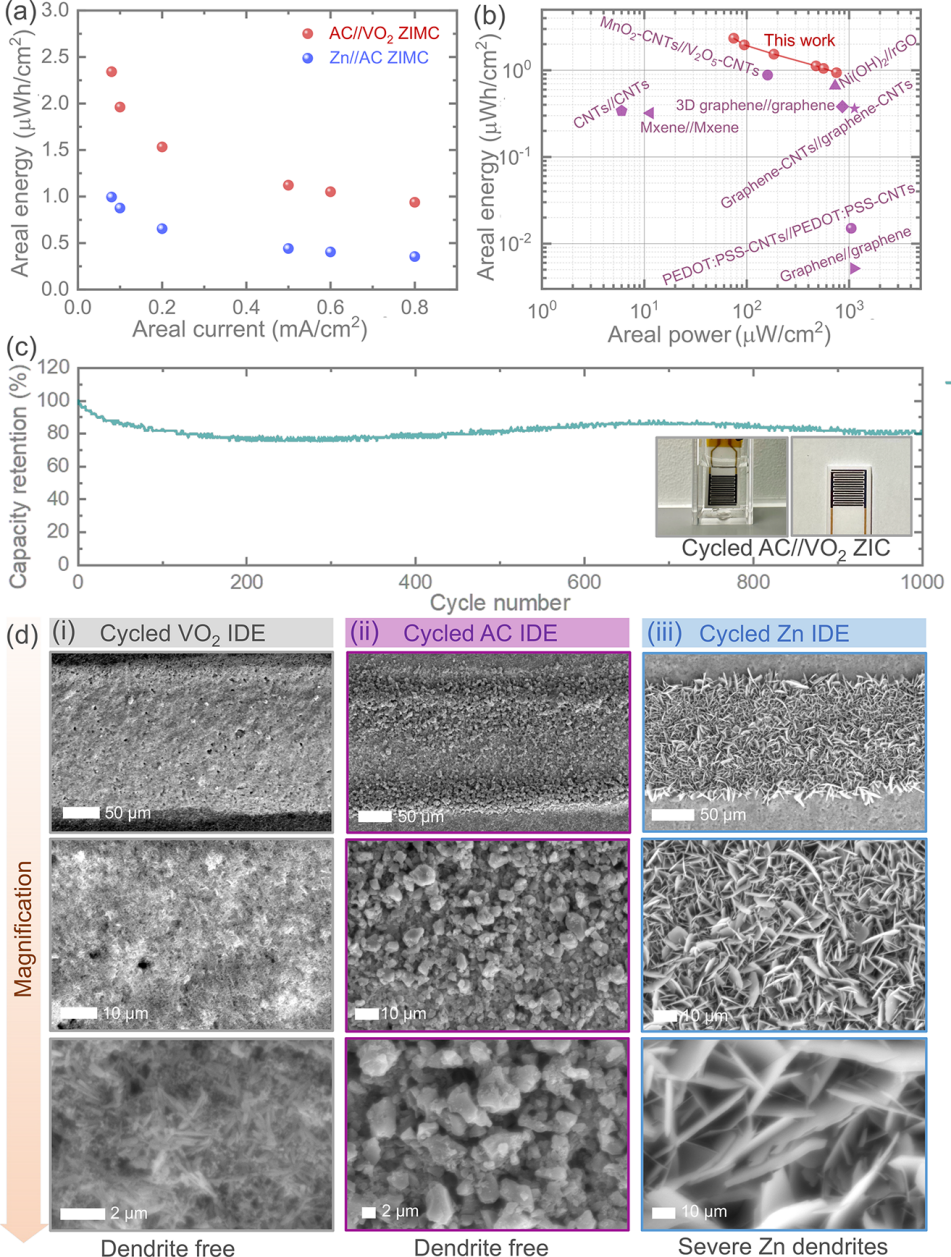
(a) Comparative areal energy plot concerning areal current, indicating a higher areal energy of AC//VO_2_ ZIMC compared to Zn//AC ZIMC. (b) Ragone plot offering a comparative overview of our AC//VO_2_ ZIMC with previously reported microsupercapacitors, encompassing both symmetric (graphene//graphene,^26^ PEDOT:PSS-CNT//PEDOT:PSS-CNT,^27^ CNTs//CNTs,^28^ MXene//MXene,^29^ 3D graphene//graphene,^30^ graphene-CNTs//graphene-CNT^31^) and asymmetric (MnO_2_–CNTs//V_2_O_5_–CNTs,^32^ Ni(OH)_2_//rGO^33^) designs. (c) Extended cycling response of a AC//VO_2_ ZIMC at 0.1 mA/cm^2^ for 1000 GCD cycles in 1 M ZnSO_4_ aqueous electrolyte, revealing an impressive 80% capacity retention. Insets represent the digital images of the cycled ZIMC device. (d) Post-mortem SEM images of cycled electrodes: (i) VO_2_ (B), (ii) AC, and (iii) Zn nanoparticles. Interestingly, Zn nanoparticles electrodes exhibit significant flakes growth as expected, while VO_2_ (B) and AC electrodes maintain similar material morphologies as before cycling.

In summary, this study revolves around the advancement of high-performance planar ZIMCs achieved by exploiting an AC anode and VO_2_ (B) cathode, coupled with effective loading onto microelectrodes using the Microplotter technique. The combination of a high-capacity supercapacitor-type AC anode and a battery-type VO_2_ (B) cathode exhibits remarkable charge storage performance. This includes an enhanced peak areal energy of 2.34 μWh/cm^2^ and an areal power of 753.12 μW/cm^2^, surpassing Zn//AC ZIMCs (areal energy of 1.25 μWh/cm^2^). Furthermore, it ensures dendrite-free performance even during long-term cycling of 1000 cycles and capacity retention of 87%. This research opens avenues for the exploration of advanced and high-resolution Microplotter techniques. These techniques enable precise loading of unrestricted high-capacity electrodes onto confined microelectrodes, thereby realizing high-performance planar microscale energy storage devices.

## Supporting Information Available

Experimental Section; SEM images of AC and VO_2_ (B) electrodes (Figure S1); SEM image, BET isotherm plot, XRD pattern, and Raman spectrum of AC particles (Figure S2); SEM images, XRD and XPS pattern of Zn nanoparticles (Figure S3); Photographic representation and top-view SEM image of the Zn//AC ZIMC (Figure S4a, b); SEM images of the Zn nanoparticles anode and AC cathode in Zn//AC ZIMC (Figure S4c, d); Intercalation of Zn^2+^ in VO_2_ (B) projected along the b and c directions, CVs, GCD curves and extended cycling test of the VO_2_ (B) cathode tested against Zn metal anode (Figure S5); Digital image of Zn//AC and AC//VO_2_ ZIMCs immersed in gel electrolyte (Figure S6); Comparative CVs and Nyquist plots of the Zn//AC and AC//VO_2_ ZIMCs (Figure S7); Prolonged cycling performance of the ZIMCs (Figure S8) and SEM image depicting a cycled AC//VO_2_ ZIMC tested in a gel electrolyte (Figure S9). (PDF)

## References

[ref1] Li P.; Liao M.; Li J.; Ye L.; Cheng X.; Wang B.; Peng H. Rechargeable Micro-Batteries for Wearable and Implantable Applications. Small Struct 2022, 3 (9), 2200058 10.1002/sstr.202200058.

[ref2] Bounor B.; Seenath J. S.; Patnaik S. G.; Bourrier D.; Tran C. C. H.; Esvan J.; Weingarten L.; Descamps-Mandine A.; Rochefort D.; Guay D.; Pech D. Low-Cost Micro-Supercapacitors Using Porous Ni/MnO2 Entangled Pillars and Na-Based Ionic Liquids. Energy Storage Mater. 2023, 63, 102986 10.1016/j.ensm.2023.102986.

[ref3] Boruah B. D. Roadmap of In-Plane Electrochemical Capacitors and Their Advanced Integrated Systems. Energy Storage Mater. 2019, 21, 219–239. 10.1016/j.ensm.2019.06.012.

[ref4] Ding J.; Hu W.; Paek E.; Mitlin D. Review of Hybrid Ion Capacitors: From Aqueous to Lithium to Sodium. Chem. Rev. 2018, 118 (14), 6457–6498. 10.1021/acs.chemrev.8b00116. 29953230

[ref5] Sun Z.; Chu S.; Jiao X.; Li Z.; Jiang L. Research Progress of Carbon Cathode Materials for Zinc-Ion Capacitors. J. Energy Storage 2024, 75, 109571 10.1016/j.est.2023.109571.

[ref6] Liu Y.; Wu L. Recent Advances of Cathode Materials for Zinc-Ion Hybrid Capacitors. Nano Energy 2023, 109, 108290 10.1016/j.nanoen.2023.108290.

[ref7] Sui D.; Wu M.; Shi K.; Li C.; Lang J.; Yang Y.; Zhang X.; Yan X.; Chen Y. Recent Progress of Cathode Materials for Aqueous Zinc-Ion Capacitors: Carbon-Based Materials and Beyond. Carbon N Y 2021, 185, 126–151. 10.1016/j.carbon.2021.08.084.

[ref8] Sun G.; Yang H.; Zhang G.; Gao J.; Jin X.; Zhao Y.; Jiang L.; Qu L. A Capacity Recoverable Zinc-Ion Micro-Supercapacitor. Energy Environ. Sci. 2018, 11 (12), 3367–3374. 10.1039/C8EE02567C.

[ref9] Zhang P.; Li Y.; Wang G.; Wang F.; Yang S.; Zhu F.; Zhuang X.; Schmidt O. G.; Feng X. Zn-Ion Hybrid Micro-Supercapacitors with Ultrahigh Areal Energy Density and Long-Term Durability. Adv. Mater. 2019, 31 ( (3), ). 10.1002/adma.201806005. 30480352

[ref10] Yu H.; Li Q.; Liu W.; Wang H.; Ni X.; Zhao Q.; Wei W.; Ji X.; Chen Y.; Chen L. Fast Ion Diffusion Alloy Layer Facilitating 3D Mesh Substrate for Dendrite-Free Zinc-Ion Hybrid Capacitors. Journal of Energy Chemistry 2022, 73, 565–574. 10.1016/j.jechem.2022.06.028.

[ref11] Fu Q.; Hao S.; Zhang X.; Zhao H.; Xu F.; Yang J. All-Round Supramolecular Zwitterionic Hydrogel Electrolytes Enabling Environmentally Adaptive Dendrite-Free Aqueous Zinc Ion Capacitors. Energy Environ. Sci. 2023, 16 (3), 1291–1311. 10.1039/D2EE03793A.

[ref12] Sun M.; Zhang Z.; Chen J.; Zhang Y.; Wang R.; Mu H.; Lian C.; Wang W.; Wang G. Zincophile Channel Adjustment Realizes Dendrite-Free Zinc Anode for Low-Temperature Zinc-Ion Capacitors. Chemical Engineering Journal 2023, 474, 145658 10.1016/j.cej.2023.145658.

[ref13] Ma X.; Wang J.; Wang X.; Zhao L.; Xu C. Aqueous V2O5/Activated Carbon Zinc-Ion Hybrid Capacitors with High Energy Density and Excellent Cycling Stability. Journal of Materials Science: Materials in Electronics 2019, 30 (6), 5478–5486. 10.1007/s10854-019-00841-z.

[ref14] Li X.; Ma Y.; Yue Y.; Li G.; Zhang C.; Cao M.; Xiong Y.; Zou J.; Zhou Y.; Gao Y. A Flexible Zn-Ion Hybrid Micro-Supercapacitor Based on MXene Anode and V2O5 Cathode with High Capacitance. Chemical Engineering Journal 2022, 428, 130965 10.1016/j.cej.2021.130965.

[ref15] Ma X.; Cheng J.; Dong L.; Liu W.; Mou J.; Zhao L.; Wang J.; Ren D.; Wu J.; Xu C.; Kang F. Multivalent Ion Storage towards High-Performance Aqueous Zinc-Ion Hybrid Supercapacitors. Energy Storage Mater. 2019, 20, 335–342. 10.1016/j.ensm.2018.10.020.

[ref16] Wang S.; Wang Q.; Zeng W.; Wang M.; Ruan L.; Ma Y. A New Free-Standing Aqueous Zinc-Ion Capacitor Based on MnO2–CNTs Cathode and MXene Anode. Nanomicro Lett. 2019, 11 (1), 70 10.1007/s40820-019-0301-1. 34138022 PMC7770692

[ref17] Chen Q.; Jin J.; Kou Z.; Liao C.; Liu Z.; Zhou L.; Wang J.; Mai L. Zn ^2+^ Pre-Intercalation Stabilizes the Tunnel Structure of MnO _2_ Nanowires and Enables Zinc-Ion Hybrid Supercapacitor of Battery-Level Energy Density. Small 2020, 16 ( (14), ). 10.1002/smll.202000091. 32174015

[ref18] Wang Q.; Wang S.; Li J.; Ruan L.; Wei N.; Huang L.; Dong Z.; Cheng Q.; Xiong Y.; Zeng W. A Novel Aqueous Zinc-Ion Hybrid Supercapacitor Based on TiS _2_ (De)Intercalation Battery-Type Anode. Adv. Electron Mater. 2020, 6 ( (10), ). 10.1002/aelm.202000388.

[ref19] Zeng J.; Dong L.; Sun L.; Wang W.; Zhou Y.; Wei L.; Guo X. Printable Zinc-Ion Hybrid Micro-Capacitors for Flexible Self-Powered Integrated Units. Nanomicro Lett. 2021, 13 (1), 19 10.1007/s40820-020-00546-7. PMC8187672 34138202

[ref20] Fan Z.; Jin J.; Li C.; Cai J.; Wei C.; Shao Y.; Zou G.; Sun J. 3D-Printed Zn-Ion Hybrid Capacitor Enabled by Universal Divalent Cation-Gelated Additive-Free Ti _3_ C _2_ MXene Ink. ACS Nano 2021, 15 (2), 3098–3107. 10.1021/acsnano.0c09646. 33576601

[ref21] Boruah B. D.; Nandi S.; Misra A. Layered Assembly of Reduced Graphene Oxide and Vanadium Oxide Heterostructure Supercapacitor Electrodes with Larger Surface Area for Efficient Energy-Storage Performance. ACS Appl. Energy Mater. 2018, 1 (4), 1567–1574. 10.1021/acsaem.7b00358.

[ref22] Deka Boruah B.; Mathieson A.; Park S. K.; Zhang X.; Wen B.; Tan L.; Boies A.; De Volder M. Vanadium Dioxide Cathodes for High-Rate Photo-Rechargeable Zinc-Ion Batteries. Adv. Energy Mater. 2021, 11 ( (13), ). 10.1002/aenm.202100115.

[ref23] Gupta G. K.; Sagar P.; Pandey S. K.; Srivastava M.; Singh A. K.; Singh J.; Srivastava A.; Srivastava S. K.; Srivastava A. In Situ Fabrication of Activated Carbon from a Bio-Waste Desmostachya Bipinnata for the Improved Supercapacitor Performance. Nanoscale Res. Lett. 2021, 16 (1), 85 10.1186/s11671-021-03545-8. 33987738 PMC8119520

[ref24] Ding J.; Du Z.; Gu L.; Li B.; Wang L.; Wang S.; Gong Y.; Yang S. Ultrafast Zn ^2+^ Intercalation and Deintercalation in Vanadium Dioxide. Adv. Mater. 2018, 30 ( (26), ). 10.1002/adma.201800762. 29761561

[ref25] Zhang X.; Pei Z.; Wang C.; Yuan Z.; Wei L.; Pan Y.; Mahmood A.; Shao Q.; Chen Y. Flexible Zinc-Ion Hybrid Fiber Capacitors with Ultrahigh Energy Density and Long Cycling Life for Wearable Electronics. Small 2019, 15 ( (47), ). 10.1002/smll.201903817. 31609075

[ref26] Li L.; Secor E. B.; Chen K.; Zhu J.; Liu X.; Gao T. Z.; Seo J. T.; Zhao Y.; Hersam M. C. High-Performance Solid-State Supercapacitors and Microsupercapacitors Derived from Printable Graphene Inks. Adv. Energy Mater. 2016, 6 ( (20), ). 10.1002/aenm.201600909.

[ref27] Liu W.; Lu C.; Li H.; Tay R. Y.; Sun L.; Wang X.; Chow W. L.; Wang X.; Tay B. K.; Chen Z.; Yan J.; Feng K.; Lui G.; Tjandra R.; Rasenthiram L.; Chiu G.; Yu A. Paper-Based All-Solid-State Flexible Micro-Supercapacitors with Ultra-High Rate and Rapid Frequency Response Capabilities. J. Mater. Chem. A Mater. 2016, 4 (10), 3754–3764. 10.1039/C6TA00159A.

[ref28] Kim H.; Yoon J.; Lee G.; Paik S.; Choi G.; Kim D.; Kim B.-M.; Zi G.; Ha J. S. Encapsulated, High-Performance, Stretchable Array of Stacked Planar Micro-Supercapacitors as Waterproof Wearable Energy Storage Devices. ACS Appl. Mater. Interfaces 2016, 8 (25), 16016–16025. 10.1021/acsami.6b03504. 27267316

[ref29] Zhang C.; McKeon L.; Kremer M. P.; Park S.-H.; Ronan O.; Seral-Ascaso A.; Barwich S.; Coileáin C. Ó.; McEvoy N.; Nerl H. C.; Anasori B.; Coleman J. N.; Gogotsi Y.; Nicolosi V. Additive-Free MXene Inks and Direct Printing of Micro-Supercapacitors. Nat. Commun. 2019, 10 (1), 1795 10.1038/s41467-019-09398-1. 30996224 PMC6470171

[ref30] Zhang L.; DeArmond D.; Alvarez N. T.; Malik R.; Oslin N.; McConnell C.; Adusei P. K.; Hsieh Y.; Shanov V. Flexible Micro-Supercapacitor Based on Graphene with 3D Structure. Small 2017, 13 ( (10), ). 10.1002/smll.201603114. 28054423

[ref31] Bellani S.; Petroni E.; Del Rio Castillo A. E.; Curreli N.; Martín-García B.; Oropesa-Nuñez R.; Prato M.; Bonaccorso F. Scalable Production of Graphene Inks via Wet-Jet Milling Exfoliation for Screen-Printed Micro-Supercapacitors. Adv. Funct Mater. 2019, 29 ( (14), ). 10.1002/adfm.201807659.

[ref32] Yun J.; Lim Y.; Lee H.; Lee G.; Park H.; Hong S. Y.; Jin S. W.; Lee Y. H.; Lee S.; Ha J. S. A Patterned Graphene/ZnO UV Sensor Driven by Integrated Asymmetric Micro-Supercapacitors on a Liquid Metal Patterned Foldable Paper. Adv. Funct Mater. 2017, 27 ( (30), ). 10.1002/adfm.201700135.

[ref33] Huang G.-W.; Li N.; Du Y.; Feng Q.-P.; Xiao H.-M.; Wu X.-H.; Fu S.-Y. Laser-Printed In-Plane Micro-Supercapacitors: From Symmetric to Asymmetric Structure. ACS Appl. Mater. Interfaces 2018, 10 (1), 723–732. 10.1021/acsami.7b15922. 29243912

