# Supplementary material for: Planar Zn-Ion Microcapacitors with High-Capacity Activated Carbon Anode and VO2 (B) Cathode
Source: Nano Lett. 2024 Aug 20;24(35):10874–82. doi: 10.1021/acs.nanolett.4c02539 (PMC11378291; doi:10.1021/acs.nanolett.4c02539)
Supplement: Supplementary file 1 — nl4c02539_si_001.pdf [file nl4c02539_si_001.pdf]

Supporting Information

for

Planar Zn-ion Micro-capacitors with High-capacity Activated Carbon Anode and VO_2 (B) Cathode

Yujia Fan,[1] Iman Pinnock,[1] Xueqing Hu,[1] Tianlei Wang,[2] Yinan Lu,[1] Ruixiang Li,[3] Mingqing Wang,[1] Ivan P. Parkin,[2] Michael De Volder,[4] Buddha Deka Boruah[1,*]

[1]Institute for Materials Discovery, University College London, London WC1E 7JE, United Kingdom

[2]Department of Chemistry, University College London, London, WC1H 0AJ, UK

[3]School of Engineering and Materials Science, Queen Mary University of London, London, E1 4NS, UK

[4]Institute for Manufacturing, University of Cambridge, Cambridge, CB3 0FS, UK

Corresponding author: Dr. Buddha Deka Boruah

Email: b.boruah@ucl.ac.uk

Experimental Section

Materials: The raw materials for VO_2(B) synthesis were vanadium oxide received from Sigma-Aldrich and oxalic acid from Scientific Laboratory Supplies. The zinc nanopowder with an average particle size from 40 to 60 nm and triethylene glycol monomethyl ether (TEGMME) were supplied from Sigma-Aldrich. The activated carbon and Super-P conductive carbon black were originated from MTI Corporation. The poly(vinylidene fluoride) powder was obtained from Alfa Aesar, and corresponding solvent dimethyl formamide was sourced from Severn Biotech Ltd.,. It's important to note that all these chemicals were utilized without the need for additional purification.

Synthesis of VO_2: To synthesize VO_2, 1.2 g of V_2O_5 and 1.8 g of $H_2C_2O_4 \cdot 2H_2O$ were added into 40 mL of deionized water. After magnetic stirring at 75°C, a dark blue dispersion was obtained. Then, the above dispersion was transferred into a 150 mL Teflon-lined autoclave and held at 180 °C for 3 days. Finally, the VO_2 powder was obtained by washing with ethanol and deionized water, allowed by vacuum oven drying overnight at 70°C.

VO_2 electrode formation for coin cell tests: For the preparation of the cathode, the active VO2 (B) material was mixed with carbon black (EQ-Lib-SuperP) and polyvinylidene fluoride (PVDF), at a ratio of 7:2:1 respectively, with N-Methyl-2-pyrrolidone (NMP) (Sigma-Aldrich, >=99%) as the solvent. PVDF was dissolved in NMP and used in this form. Initially VO_2 and carbon black were mixed in a Thinky Mixer for 2 minutes, three times, at 2000 rpm. Next the PVDF in NMP was added drop wise and mixed again for 2 minutes, 3 times. Lastly NMP was added 100 μL at a time and mixed for 2 minutes, which was repeated until the desired slurry viscosity was obtained.

Carbon paper was used as the current collector; the slurry was casted on the carbon paper using a doctor blade at a thickness of 150 μm, which was then dried for 30 minutes on a hot plate at 50 °C. The casted cathodes were then transferred into the freeze dryer (Labconco FreeZone 2.5) and dried overnight. The dried cathodes were cut into 14 mm circles.

Coin cell (CR2032) were assembled by first placing the formed cathode in the center of the case, then placing a 19 mm diameter Whatman glass microfiber filter on top and adding 150 μL of 3M $Zn(CF_3SO_3)_2$ (Sigma-Aldrich) aqueous electrolyte dropwise. Zinc foil (0.07 mm thick) was cut into 14 mm diameter circles and used as the anode, which was placed in the

center on the separator, followed by 1mm thick spacer and 1.1 mm thick spring. The base of the cell was placed on top and crimped to 750 psi, to complete the cell.

Initially, CV curves were tested over a working voltage range of 0.2 to 1.6 V at different scan rates, ranging from 0.1 to 1 mV s^{-1}, using a Biologic MPG-2 battery testing system. Furthermore, galvanostatic charge-discharge measurements were taken at different specific currents, ranging from 100 to 10,000 mA g^{-1}. Long term cycling of the cells was measured, at specific currents of 1000 mA g^{-1}, using a Neware battery tester.

Preparation of electrode inks: For electrode inks, 10 wt% PVDF in DMF as binder and Super-P carbon black as conductive additive were added in the ink. The detailed weight ratio of active material, conductive additive and binder was 88:10:2, corresponding to 0.88 g of zinc nanopowder, VO_2 powder or activated carbon powder, 0.1 g of Super-P carbon black and 0.2 g of 10 wt% PVDF in DMF solution. Then, 4 g, 5 g and 5.5 g of TEGMME were added into zinc electrode powder mixture, VO_2 electrode powder mixture and activated carbon electrode mixture, respectively. After tip sonicating for 30 minutes of each mixture by Fisherbrand 505 sonciator, the electrode inks were obtained. The amplitude and pulse configuration of sonicator were set as 30% and 0101, separately.

Printing of ZIMCs: The allow printing process was conducted by SonoPlot Microplotter Proto with a 20 μm nozzle. Before printing, gold interdigitated electrodes (IDEs) with 200 μm gaps from Metrohm UK Ltd., were immersed in acetone to pre-clean the surface, the pattern of ZIMCs was designed by using SonoDraw software. During printing, the nozzle was aligned with the starting point of IDE pattern, the feature size and printing mode were set as 50 μm and spraying mode. The printing voltages applied were 8 V for zinc electrode ink, 10 V for VO_2 electrode ink and 12 V for activated carbon electrode ink.

Material Characterization: Scanning electron microscopy (SEM) was used to examine the morphology and microstructure of the raw materials, zinc, VO_2 and activated carbon powder, using the ZEISS Sigma 500 Field Emission Scanning Electron Microscope. Raman spectroscopy, specifically the RENISHAW inVia Raman microscope, was employed to gain insights into the crystalline information of these powders. X-ray diffraction (XRD) analysis was performed using the Malvern Panalytical Aeris X-ray diffractometer to assess the

crystalline properties of the powders. BET measurements were conducted using the NOVAtouch instrument to explore the specific surface area of the activated carbon.

A stylus profilometer, the DektakXT from Bruker, was employed to create a 3D mapping of the printed ZIMCs. XPS analysis was performed using the Thermo NEXSA XPS instrument fitted with a monochromated Al kα X-ray source, a spherical sector analyzer, and multichannel resistive plate detectors. Data was recorded at 19.2W and an X-ray beam size of 400 x 200 μm, with survey scans recorded at a pass energy of 200 eV and high-resolution scans recorded at a pass energy of 40 eV. Electronic charge neutralization was achieved using a Dual-beam low-energy electron/ion source (Thermo Scientific FG-03), and the sample data was recorded at a pressure below 10-8 Torr and a room temperature of 294 K. CasaXPS v2.3.20rev1.0o was used for data analysis.

Electrochemical testing: For the printed ZIMCs, 1M zinc sulphate ($ZnSO_4$) gel electrolyte was prepared by dissolving 2 g of gelatine into 15 mL of 1M $ZnSO_4$ electrolyte at 80°C. During assembly of ZIMCs, two pieces of copper foil were adhered to the electrode ends using silver paste, and the junctions were covered with Kapton tape. Then, the ZIMC was placed into a cuvette (Fisherbrand Disposable Cuvettes 14955125), and the gel electrolyte was introduced into the cuvette. Finally, the top of the cuvette was sealed by parafilm, and the extension of copper foil was fixed on the outer wall of the cuvette.

The electrochemical measurement of printed ZIMCs was obtained by various testers. The cyclic voltammetry (CV) tests at scan rates from 10 to 500 mV/s and galvanostatic charge-discharge (GCD) tests at areal current densities from 0.08 mA/cm^2 to 10 mA/cm^2 were conducted on a battery testing system (MPG2, BioLogic). Meanwhile, long-term cycling at 0.1 mA/cm^2 for 1000 cycles was conducted by Neware testers, electrochemical impedance spectroscopy (EIS) tests were carried out within a frequency range from 10 mHz to 100 kHz at a voltage amplitude of 10 mV by using IVIUM Potentiostat/Galvanostat tester.

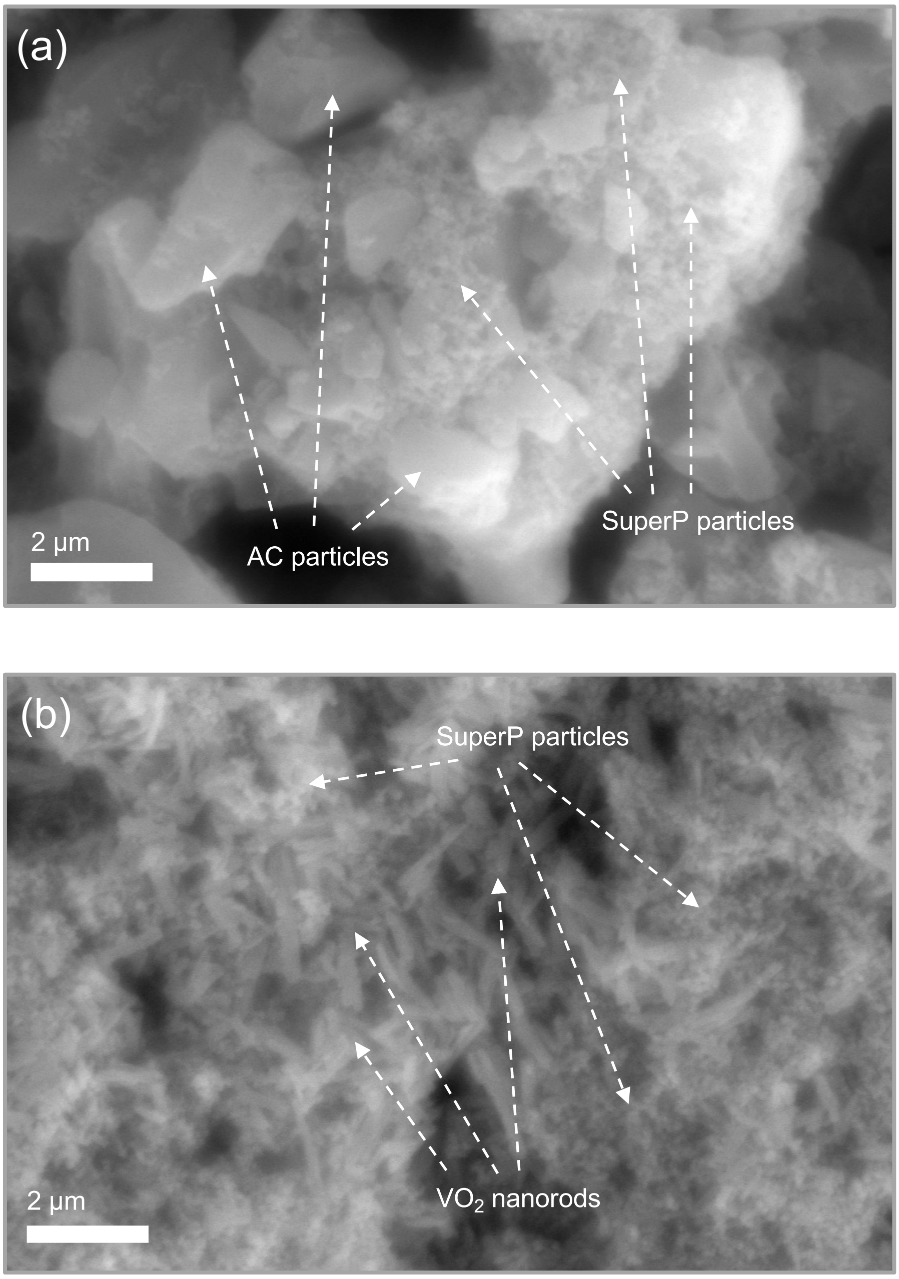

Figure S1. *SEM images of (a) AC anode and (b) VO_2 cathode.*

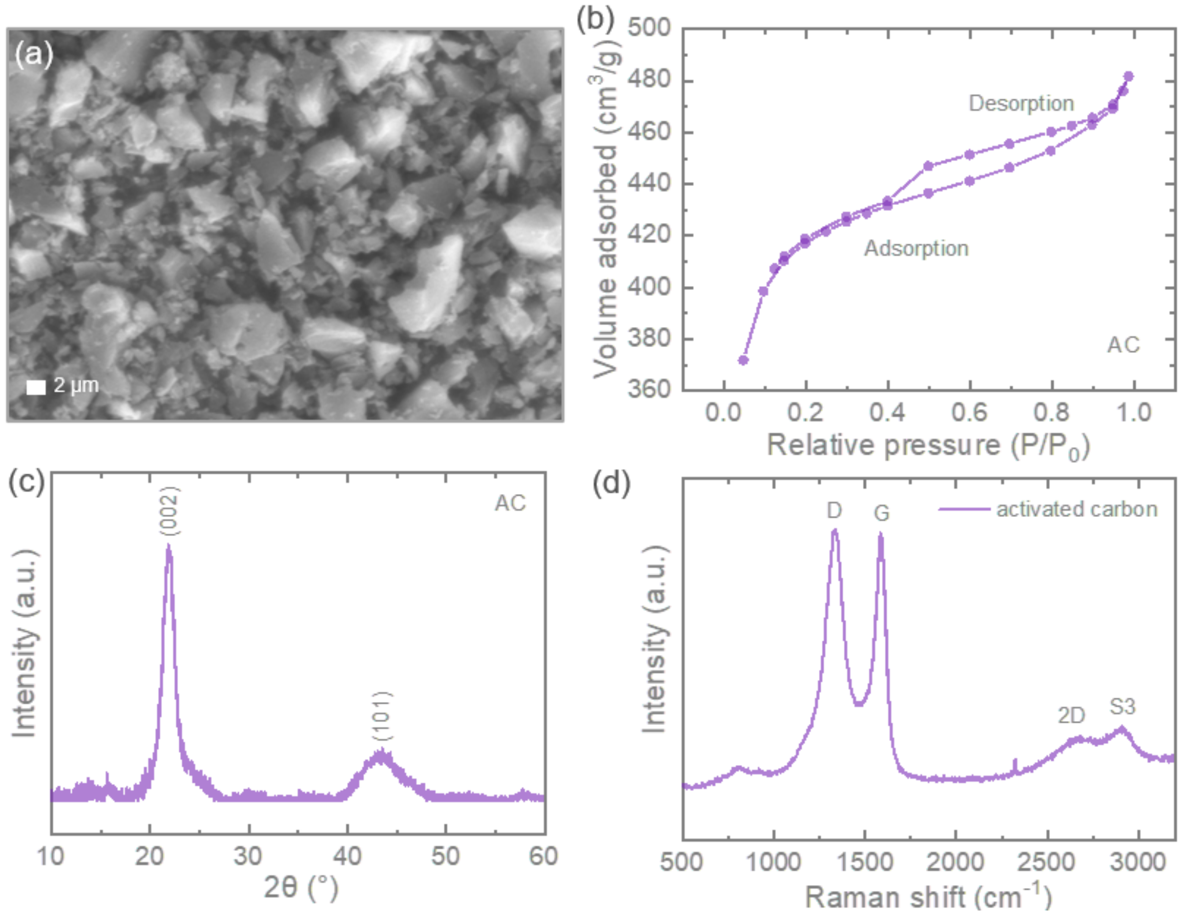

Figure S2. *(a) SEM image of activated carbon powder, (b) BET isotherm plot, (c) XRD pattern, and (d) Raman spectrum of AC particles.*

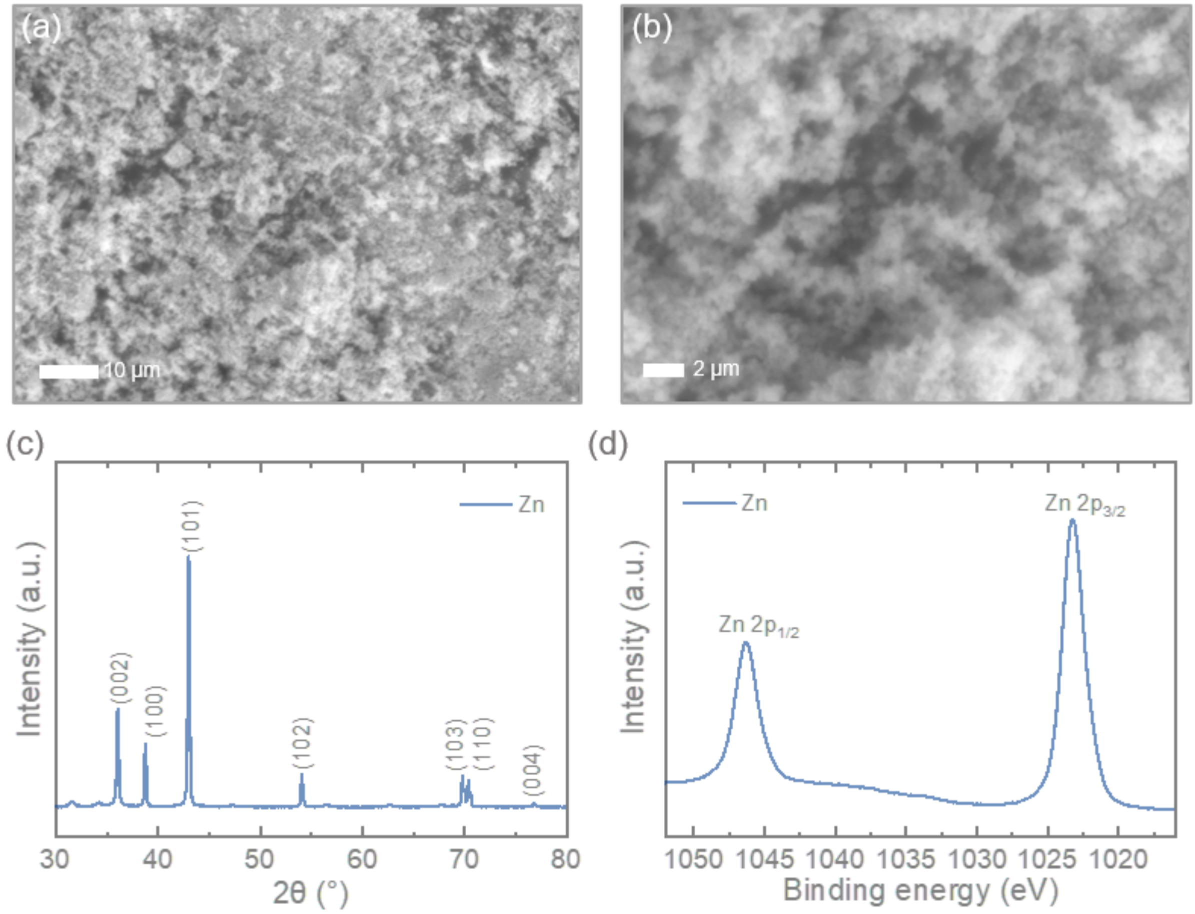

Figure S3. *(a, b) SEM images of zinc powder, (c) XRD pattern of Zn nanoparticles, and (d) XPS spectra of Zn 2p.*

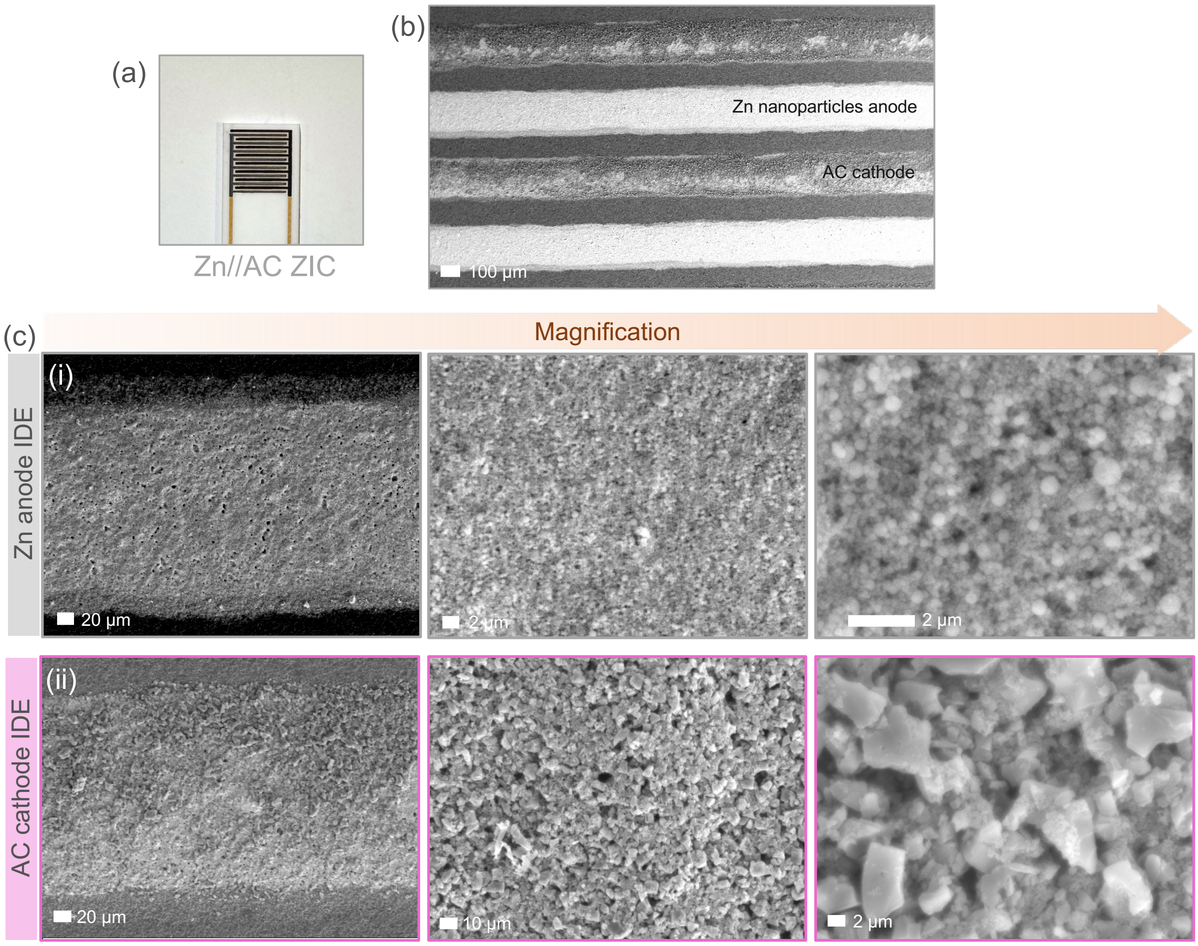

Figure S4. *(a) Photographic representation of a Zn//AC ZIMC. (b) Top-view SEM image of the Zn//AC ZIMC. (c) SEM images illustrating (i) the Zn nanoparticles anode and (ii) the AC cathode at various magnifications.*

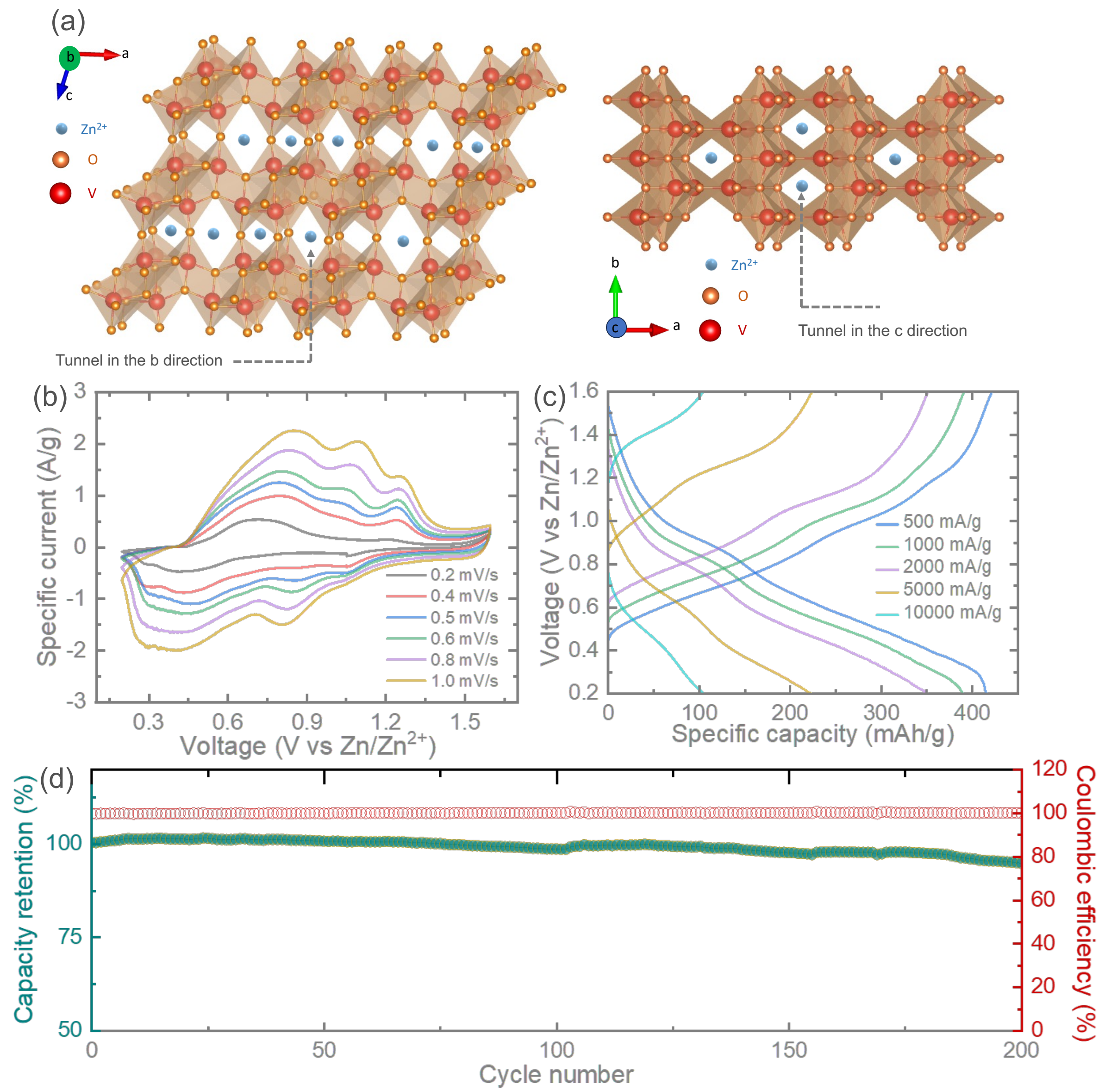

Figure S5. *(a) Schematic illustration depicting the intercalation of* Zn^{2+} *in* VO_2 *(B) projected along the b and c directions. (b, c) CVs and GCD curves of the* VO_2 *cathode tested against Zn metal anode in a coin cell-based Zn-ion battery configuration. (d) Extended cycling test (post-activation) of the* VO_2 *cathode tested against Zn metal anode at 2000 mA/g.*

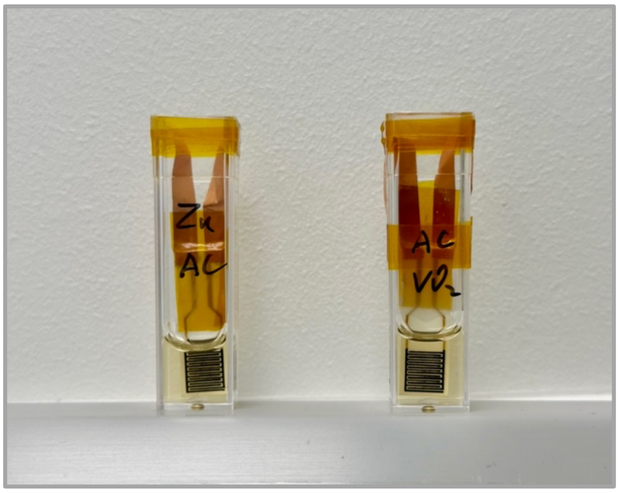

Figure S6. *Photographic documentation of Zn//AC and AC//VO_2 ZIMCs immersed in gel electrolyte during testing.*

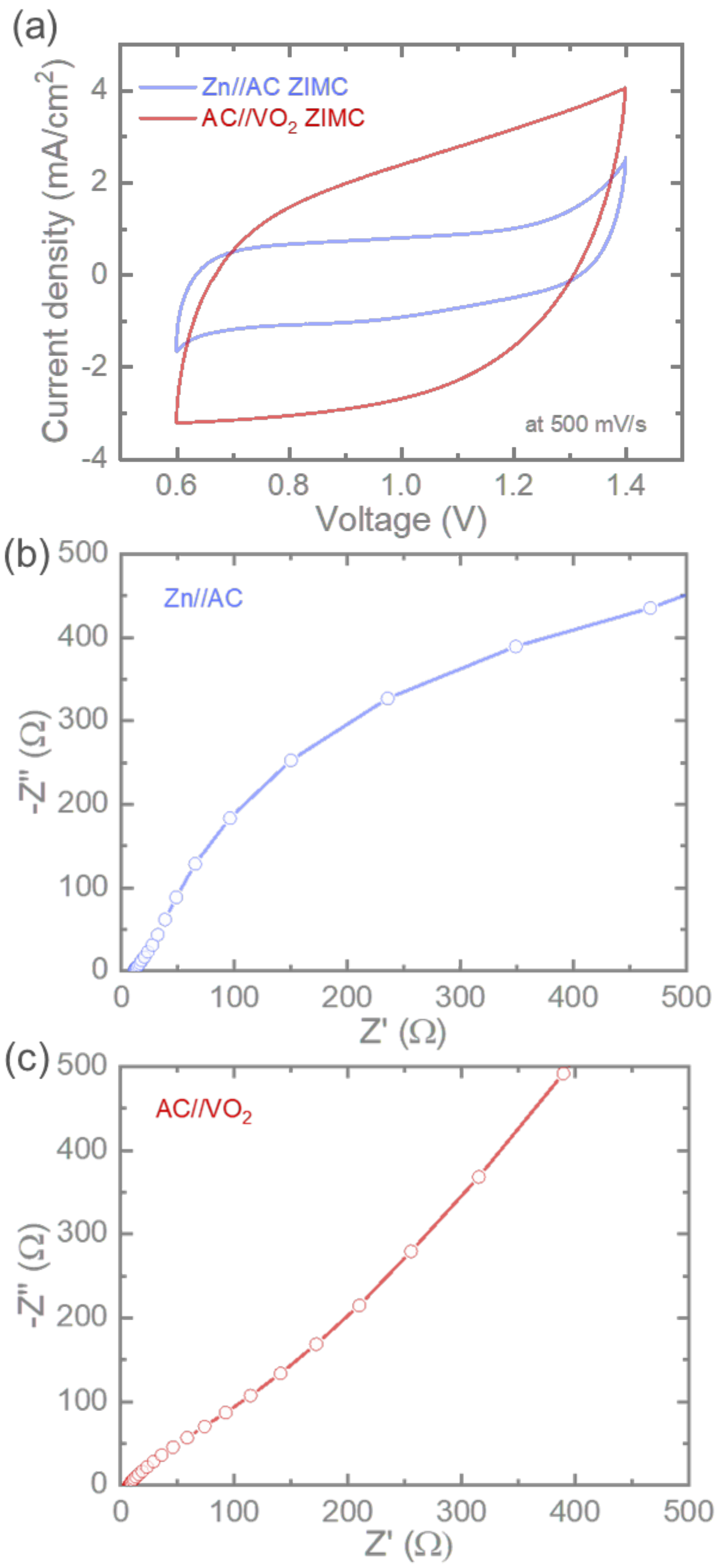

Figure S7. *(a) Comparative CVs of the Zn//AC and AC//VO_2 ZIMCs tested at 500 mV/s. Nyquist plots of (b) Zn//AC ZIMC and (c) AC//VO_2 ZIMC.*

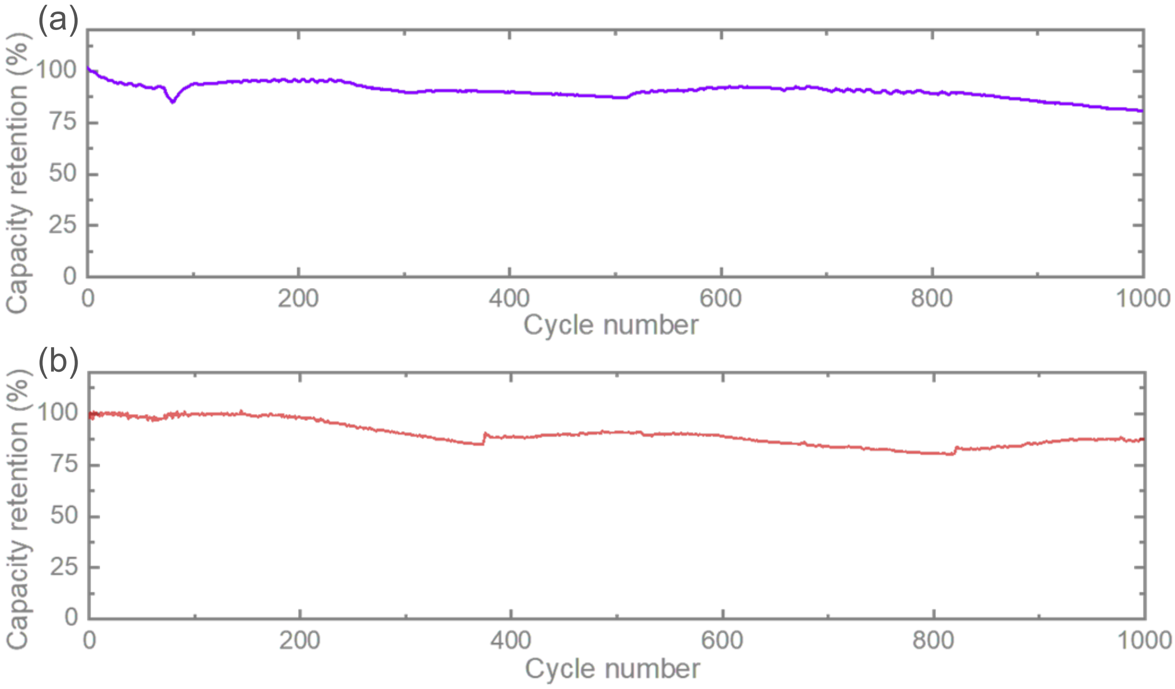

Figure S8. *Prolonged cycling performance of (a) Zn//AC and (b) AC//VO_2 ZIMCs at 0.1 mA/cm², examined in a gel electrolyte.*

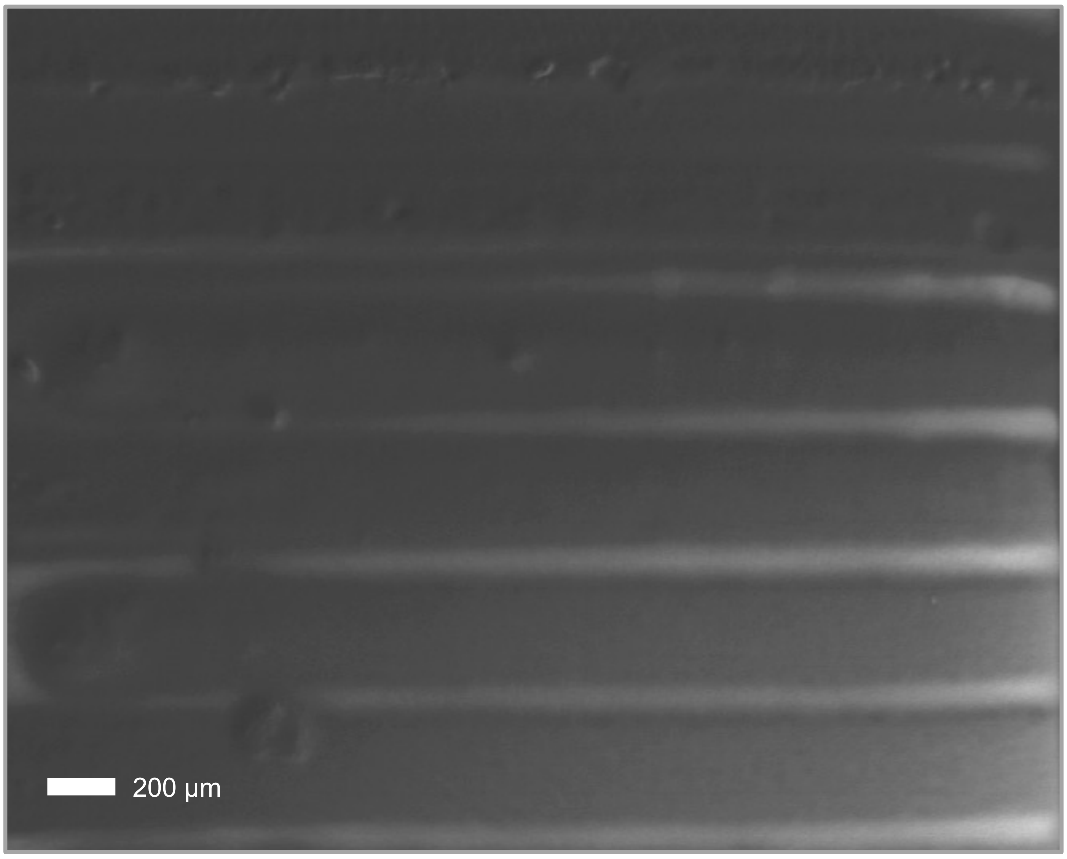

Figure S9. *SEM image depicting a cycled AC//VO_2 ZIMC tested in a gel electrolyte.*
